# Combined CDK4/6 Inhibition and Radiation: Effects on Cellular Senescence, Cell Cycle Regulation, and Cell Death in Mammary Carcinoma Cells

**DOI:** 10.3390/cells15080734

**Published:** 2026-04-21

**Authors:** Lisa Quarz, Luitpold V. Distel, Stefanie Corradini, Laura S. Hildebrand

**Affiliations:** 1 Department of Radiation Oncology, Universitätsklinikum Erlangen, Friedrich-Alexander-Universität Erlangen-Nürnberg (FAU), Universitätsstraße 27, 91054 Erlangen, Germany; lisa.quarz@fau.de (L.Q.); laura.hildebrand@uk-erlangen.de (L.S.H.); 2 Comprehensive Cancer Center Erlangen-Europäische Metropolregion Nürnberg (CCC ER-EMN), 91054 Erlangen, Germany

**Keywords:** CDK inhibition, senescence, apoptosis, necrosis, cell cycle arrest, palbociclib, ribociclib, abemaciclib, ionizing radiation, breast cancer cell lines

## Abstract

**Highlights:**

**What are the main findings?**

- CDK4/6 inhibitors induce senescence in many breast cancer cells, especially in MCF-7, HTB-133, MDA-MB-231 and BT-20.
- Cell death in the form of apoptosis or necrosis was not a predominant cell fate following CDK inhibition.
- Combination therapy with CDK inhibition and irradiation showed no clear additive effect.

**What are the implications of the main findings?**

- Senescence predominates over cell death after CDK4/6 inhibition, suggesting that cell inactivation is primarily due to senescence rather than cell death.
- Examination of the proliferation marker Ki-67 suggests a therapeutic window, enabling effective tumor cell treatment without increased damage to healthy tissue.

**Abstract:**

CDK4/6 inhibitors such as palbociclib, ribociclib and abemaciclib are commonly used in the clinical treatment of HR-positive, HER2-negative metastatic or locally advanced breast cancer. Patients with metastatic disease often receive palliative radiotherapy for symptom control of bone metastases and/or local lesions, typically administered in close temporal proximity to CDK4/6 inhibitor therapy, although treatment with the inhibitors may be temporarily paused during the radiotherapy period in some cases. In this study, we investigated the extent to which senescence is induced by CDK4/6 inhibitors, ionizing radiation, and the combination of the two, compared to other types of cell fate. Eight breast cancer cell lines with different molecular subtypes and two healthy cell lines (fibroblasts and keratinocytes) were treated with CDK inhibition using palbociclib, ribociclib or abemaciclib and with or without a single dose of 2 Gy ionizing radiation. Cellular senescence, cell death in form of apoptosis and necrosis, and the cell cycle were analyzed using flow cytometry. We focused mainly on understanding how CDK inhibition can trigger cellular senescence. Our data showed that in many cell lines —but not all—the use of CDK inhibitors induced senescence much more strongly than cell death. Except for one cell line, significantly more cell lines died necrotically than apoptotically. Neither apoptosis nor necrosis was responsible for a major cell fate after CDK inhibition. Combination therapy with irradiation did not show a clear additive effect. In cell lines, senescence is clearly triggered by CDK4/6 inhibitors and even more so when in combination with ionizing radiation, which, when transferred to patients, could lead to less damage caused by cell loss, such as necrotic areas. However, it could also lead to more senescence-specific side effects, such as inflammation-induced tumors and fibrosis.

## 1. Introduction

Cyclin-dependent kinase (CDK) 4/6 inhibitors are now an established treatment option for patients with hormone receptor (HR)-positive, human epithelial receptor 2 status (HER2)-negative, metastatic or locally advanced breast cancer [1,2]. The administration of treatment is invariably accompanied by hormone therapy due to the fact that cyclin D is estrogen-dependent [1]. In clinical practice, this approach is often employed in conjunction with radiation therapy, particularly for the management of symptoms related to bone metastases [3]. To date, there is a paucity of clinical research on the subject of combination therapy, as evidenced by the recent literature [1].

The available preclinical data suggest that the combination of CDK4/6 inhibition and irradiation may have a synergistic effect [1,2,4]. Ionizing radiation has been observed to cause direct and indirect damage to DNA, which in the absence of adequate DNA repair, can result in cellular death [1]. The hypothesis of a synergistic effect is based on the premise that CDK4/6 inhibitors impede the cell cycle in the G1 phase, while radiation hinders cell cycle progression in the G2-M phase [1]. Another potential cause of the synergistic effect could be the promotion of apoptosis by CDK4 inhibitors [4].

Cellular senescence is defined as a state in which the active division of cells, otherwise known as cell proliferation, is impeded [5,6]. Initially, therefore, an inhibitory effect on tumor growth was assumed and expected [6]. Nevertheless, clinical studies have also observed the opposite effect, namely an increase in tumor growth due to senescence [5,6]. Senescence manifests itself as a change in cell metabolism, visible, among other things, in the expression of cytokines, chemokines, growth factors, and proteins [5,6]. This change in metabolism in its entirety is collectively referred to as the senescence-associated secretory phenotype (SASP) [5,6]. In the short term, an acute tumor-suppressive effect is currently described, as in particular T cells and NK cells are stimulated to eliminate cells [5]. In contrast, long-term, chronic SASP expression has been shown to have a significant effect on the development of malignant tumors, presumably due to a pro-inflammatory and immunosuppressive effect [5].

Senescence can be triggered by a wide variety of cell-damaging stimuli, such as ionizing radiation or oncogenic signals [5]. Oncogene-induced senescence (OIS) is an important protective mechanism of tumor suppression, resulting in a stable arrest in the cell cycle so that malignant degenerated cells cannot proliferate [5]. Activation occurs via various growth factors (e.g., Epidermal Growth Factor), which cause metabolic intracellular changes via different signaling pathways [5]. Our main focus was on understanding how the above-mentioned CDK4/6 inhibitors trigger cell death compared to cellular senescence, along with radiation in eight distinct breast cancer cell lines and two normal tissue cell lines.

## 2. Materials and Methods

### 2.1. Cell Lines

A total of ten cell lines have been used, including eight breast cancer cell lines (MCF-7, HTB-133, HTB-20, HTB-30, MDA-MB-231, HTB-132, BT-20, BT-549) and two non-cancerous cell lines (SBLF-9 and HaCaT). MCF-7, MDA-MB-231, BT-549 and BT-20 were purchased from CLS Cell Lines Service (Eppelheim, Germany). HTB-133, HTB-20, HTB-132 and HTB-30 were kindly provided by Matthias Rübner (Department of Gynecology, University Hospital Erlangen). The human-skin fibroblasts SBLF-9 were derived from a healthy 73-old donor [A document indicating the agreement of the Ethics Committee of the medical faculty of the Friedrich-Alexander-Universität Erlangen-Nürnberg (204_17 BC) on 18 August 2017 is available; an informed consent form was signed]. Healthy HaCaT keratinocytes were derived from a healthy individual and subsequently cultured. They were obtained from the Department of Ophthalmology at the University Clinic Erlangen.

Breast cancer cell lines can be classified in various ways [7,8]. The literature often refers to the molecular classification into luminal A, luminal B, HER2-positive, and triple-negative, which is also clinically relevant in the context of therapy selection [7,8,9,10]. The distinction is made on the basis of gene expression, such as estrogen receptor status (ER), progesterone receptor status (PR), and human epithelial receptor 2 status (HER2); HR-positive refers to tumors expressing ER and/or PR [7,8]. The breast cancer cell lines used in this study were classified according to this classification system (Table 1).

The panel of breast cancer cell lines included in this experiment was chosen to be broad, to enable a comparative assessment of CDK4/6 inhibitor-induced senescence across different molecular backgrounds. This approach was selected to explore whether senescence induction is largely confined to the clinically targeted HR-positive or HER2-negative subtype or can also occur in other subtypes, thereby providing a broader mechanistic context for our findings.

### 2.2. Cell Culture

All cells were cultured in different specialized medium during the proliferation process, we supplemented each medium with 10% non-inactivated Fetal Bovine Serum (FBS) (Sigma-Aldrich, St. Louis, MO, USA) and 1% penicillin–streptomycin (Gibco, Waltham, MA, USA) unless stated otherwise.

We used Dulbecco’s Modified Eagle’s Medium (DMEM) (PAN Biotech, Aidenbach, Germany) supplemented as said above for MCF-7, MDA-MB-231 and BT-20. For HTB-20, we additionally added 17.8 mM sodium bicarbonate (PAN Biotech, Aidenbach, Germany), 25 µM glucose (Gibco, Waltham, MA, USA) and 20 µM L-Glutamine (Gibco, Waltham, MA, USA). HTB-30 cells were cultured in McCoy 5A-Medium (Gibco, Waltham, MA, USA) and HTB-132 in DMEM/F-12 (Gibco, Waltham, MA, USA). For HTB-133, we used RPMI-1740-Medium (Sigma-Aldrich, St. Louis, MO, USA) with added 10 µg/mL insulin (Gibco, Waltham, MA, USA). HaCaT and BT-549 were grown in DMEM with 4.5 g/L glucose (Gibco, Waltham, MA, USA). SBLF-9 cells were cultured using F12-Medium (Gibco, Waltham, MA, USA) supplemented with 15% FBS and 2% non-essential amino acids (NEA) (Bio&Sell, Nuremberg, Germany). The individual medium for every cell line ensures stable proliferation during cultivation and therefore enables the investigation of effects caused by the therapeutics used. All cells were maintained at 37 °C in a humidified atmosphere of 5% CO_2_ and split regularly; they were not used beyond passage 50. SBLF-9 fibroblasts were used between passages 5 and 15, and HaCaT keratinocytes between passages 5 and 10.

### 2.3. Combined Assay for Senescence, Cell Cycle, Apoptosis, and Necrosis by Flow Cytometry

On day 0, cells were seeded into T25 culture flasks and maintained in medium supplemented with 10% FBS, except for SBLF-9 where 15% FBS was used.

On day 1, after 24 h of incubation, the medium was replaced with the respective culture medium containing a reduced serum concentration (2% FBS).

Cells were then treated with the CDK4/6 inhibitors palbociclib, ribociclib or abemaciclib at a final concentration of 0.5 µM. Palbociclib (MW 573.7 g/mol), ribociclib (MW 434.54 g/mol) and abemaciclib (MW 602.7 g/mol) (Selleck Chemicals, Huston, TX, USA) were prepared as stock solutions with a target concentration of 1 mmol/L and stored at −80 °C. Palbociclib was dissolved in aqua bidest, while ribociclib and abemaciclib were dissolved in dimethyl sulfoxide (DMSO) (Roth, Karlsruhe, Germany). The stock solutions were diluted 1:10 in Dulbecco’s Phosphate-Buffered Saline (DPBS) (Sigma-Aldrich, St. Louis, MO, USA) for further use. In the samples treated with ribociclib or abemaciclib, this results in a final DMSO concentration of 0.05%. Required aliquots were thawed immediately prior to each experiment. The dose of CDK4/6 inhibitors were selected based on the steady-state serum concentrations measured in patients. Since higher serum concentrations are achieved with ribociclib, we chose the same dose of 0.5 µM for all three inhibitors to ensure comparability (Table 2).

**Figure 1:**
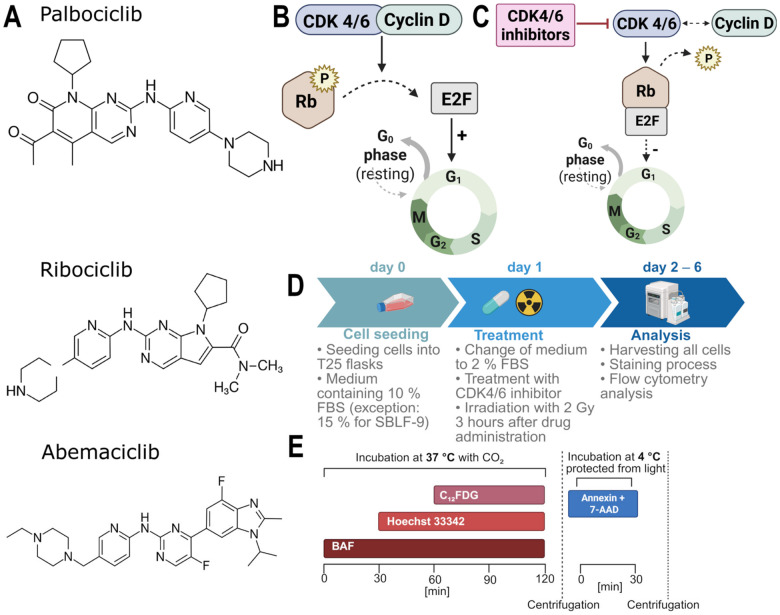
Inhibitors used, mode of action, and timeline. Chemical structures of the three used inhibitors: palbociclib, ribociclib and abemaciclib (**A**) [14]. Schematic representation of CDK4/6 kinases bound to cyclin D, which phosphorylate the pRB and release the E2F transcription factor, thus initiating the cell cycle (**B**). CDK4/6 inhibitors inhibit the kinase activity of CDK. As a result, the pRB is not phosphorylated, or only to a reduced extent, so that E2F is no longer released and the cell cycle can no longer start (**C**). Schematic overview of the experimental procedure, the arrow indicates the sequence of the individual steps (**D**) and timeline of the staining process with BAF preloading for C_12_FDG senescence staining, cell death detection using Annexin V/7-AAD, and cell cycle analysis using Hoechst 33342 (**E**). Created in BioRender. Quarz, L. (2026); https://BioRender.com/khczv1c (accessed on 27 March 2026); https://BioRender.com/e3gmsxc (accessed on 27 March 2026); https://BioRender.com/rx45817 (accessed on 27 March 2026); https://BioRender.com/i9u7935 (accessed on 27 March 2026).

For combination treatments, cells were exposed to ionizing radiation 3 h after drug administration, using an ISOVOLT Titan X-ray generator (GE, Ahrensburg, Germany) at a total dose of 2 Gy. MCF-7 and MDA-MB-231 were analyzed by flow cytometry every 24 h between 24 and 120 h after inhibitor addition. We chose to perform additional senescence measurements on the remaining cell lines 72 h after inhibitor addition, as a compromise based on the results obtained from the MCF-7 and MDA-MB-231 cell lines. A schematic overview of the experiments performed can be found in Figure 1D; Table 3 also provides a detailed overview of every day.

Control and irradiation monotherapy samples underwent the same process as described, without addition of a CDK4/6 inhibitor or substitute solution. All experiments were performed thrice.

### 2.4. Staining Process for Flow Cytometry Analysis

Both adherent and detached cells were collected for staining; Figure 1E provides a graphical summary of the dyeing process, which is explained in detail below. Cells were washed with DPBS (Sigma-Aldrich, St. Louis, MO, USA) and detached using 0.5% trypsin-EDTA (Gibco, Waltham, MA, USA). The culture medium and DPBS wash were retained to ensure inclusion of all cells. After centrifugation (8 min, 180× *g*), the supernatant was discarded, and the cells were resuspended in cell culture medium (2% FBS) with a total volume of 400 µL per sample. Afterwards, 4 µL Bafilomycin A 1 (BAF) (10 µg solved in 1.6 mL DMSO) (EMD Millipore, Merck, Darmstadt, Germany) was added to each sample before incubating them for 30 min at 37 °C in a 5% CO_2_ atmosphere.

BAF was added in preparation of the C_12_FDG-staining process to increase the pH of the lysosomes, preventing the highly active lysosomal β-galactosidase in non-senescent, healthy cells from cleaving the C_12_FDG substrate. This ensures that only the senescent-associated β-galactosidase (SA-β-Gal), which is more active at a slightly alkaline pH, can cleave the substrate and produce a green fluorescent signal, allowing for the detection of senescent cells [15].

Cells were then stained with 4 µL Hoechst 33342 (1 mg/mL; Molecular Probes, Eugene, OR, USA) per sample and incubated for an additional 30 min at 37 °C in a 5% CO_2_ atmosphere.

Following that, 0.5 µL C_12_FDG (5 mg dissolved in 300 µL DMSO) (Invitrogen, Waltham, MA, USA) was added per sample, and cells were incubated again at 37 °C in a 5% CO_2_ atmosphere for 60 min.

After another round of centrifugation (6 min, 400× *g*), cell pellets were resuspended in 200 µL of Ringer’s solution (Fresenius, Bad Homburg, Germany). Subsequently, 5 µL APC Annexin V (BD Pharmingen, Franklin Lakes, NJ, USA) and 5 µL 7-AAD (BD Pharmingen, Franklin Lakes, NJ, USA) were added to each sample and the mixtures were incubated for 30 min at 4 °C in the dark. The samples were then centrifuged again (6 min, 400× *g*), the supernatant was discarded, and the cells were resuspended in 200 µL of Ringer’s solution per sample. Finally, the samples were transferred to a 96-well plate (Greiner Bio-One, Kremsmünster, Austria) for flow cytometric analysis with the Cytoflex S (Beckman Coulter GmbH, Krefeld, Germany).

### 2.5. Flow Cytometry Analysis

Analysis of cell death, specifically apoptosis and necrosis, was performed using the previously described staining with Annexin V and 7-AAD. Necrotic cells were defined as double-positive for Annexin V and 7-AAD, whereas apoptotic cells were identified as Annexin V-positive but 7-AAD-negative. Cell cycle analysis was conducted using Hoechst 33342 staining. The distinction between S phase, G0/1 phase, and G2/M phase was determined based on DNA content. Cellular senescence was assessed using C_12_FDG staining in combination with prior treatment with BAF. C_12_FDG-positive cells were quantified both within the entire population and within the subset of viable cells. All data that were generated by flow cytometry were analyzed with Kaluza Analysis 2.3 (Beckman Coulter GmbH, Krefeld, Germany). The complete gating strategy is shown in Figure S1 of the Supplementary Material and is illustrated based on the two cell lines, BT-549 and HTB-132, as representative examples.

### 2.6. Immunofluorescence Microscopy by Immunostaining

In addition, as confirmatory visualization, cell analysis of all cell lines mentioned above was performed using immunostaining. These stainings were intended as qualitative, illustrative experiments rather than independent quantitative replicates, and were consequently performed only once. Treatment was carried out as described above, using palbociclib as the sole representative of CDK4/6 inhibitors. Palbociclib was randomly selected from the three inhibitors tested to ensure an unbiased choice. The cells were seeded directly onto coverslips on day 0, using the same media for each cell line as described above, with the commonly used FBS concentration.

On the following day, the medium was changed to a lower FBS concentration of 2%, and treatment with palbociclib at a final concentration of 0.5 µM was carried out, as well as irradiation with 2 Gy 3 h after drug application. On day 4—72 h after inhibitor addition —the evaluation was conducted. For this purpose, the medium was discarded, the cells were washed with DPBS and then fixed with formaldehyde solution (4 wt.%) (10.8 mL Formaldehyde solution 37 wt.% in H_2_O (Sigma-Aldrich, St. Louis, MO, USA), 89.2 mL 1× DPBS, 100 µL Triton X-100 (Sigma-Aldrich, St. Louis, MO, USA). Following a reaction time of 15 min on the laboratory shaker, three washing cycles were performed using 1× Tris-buffered saline (TBS) (100 mL 10× TBS, 900 mL Aqua bidest)) (10× TBS: 12.114 g TRIS (Roth, Karlsruhe, Germany), 87.6 g Sodium chloride (Roth, Karlsruhe, Germany) in 1000 mL Aqua bidest, pH = 8). The cells were subsequently placed in the blocking solution (90 mL 1× DPBS, 10 mL FBS, 0.3 mL 1 M Sodium azide (EMD Millipore, Merck, Darmstadt, Germany), 1 g Albumin bovine Fraction V (SERVA Electrophoresis GmbH, Heidelberg, Germany)) and left to incubate for a period of 1 h at ambient temperature. This was followed by three further washing cycles with 1× TBS on the laboratory shaker.

Subsequently, the application of antibodies can commence. For this purpose, we utilized the mouse antibodies anti α-tubulin (a component of microtubules) (Abcam, Cambridge, UK) and anti Ki-67 (a proliferation marker) (Santa Cruz Biotechnology, Santa Cruz, CA, USA) as primary antibodies, and the rabbit antibodies anti H3K9me3 (a histone modification) (Merck, Darmstadt, Germany) and anti p21 (a CDK inhibitor) (Cell Signaling, Danvers, MA, USA). The antibodies were applied locally to the slides. Following a reaction time of 2 h at ambient temperature in the humidity chamber, three additional washing cycles with 1× TBS were conducted on the laboratory shaker. The secondary antibodies were then applied in the same manner, with the rabbit antibodies (H3K9me3 and p21) (Invitrogen Alexa Fluor 488 chicken anti rabbit IgG, Thermo Fisher, Waltham, MA, USA) stained green and the mouse antibodies (α-tubulin and Ki-67) (Invitrogen Alexa Fluor 594 goat anti mouse IgG, Thermo Fisher, Waltham, MA, USA) stained red. Following an exposure time of 1 h at ambient temperature in the humidity chamber, three additional washing cycles were conducted using 1× TBS on the laboratory shaker. The DAPI (Sigma-Aldrich, St. Louis, MO, USA) (blue) dye was then applied to each slide, following which the slides were incubated in the dark for a period of one minute. Subsequently, a final washing process was conducted using Aqua bidest. Vectashield containing DAPI (Vector Laboratories, Mowry Ave, Newark, CA, USA) was used to cover the cells.

Images were acquired using a Zeiss Axioplan 2 fluorescence microscope (Göttingen, Germany) controlled using Metafer 4 V3.10.1 Metasystems software (Altlussheim, Germany). We marked the DAPI-stained cell nuclei using image analysis software (Biomas, V.2.1., Erlangen, Germany) and measured their brightness for p21 and Ki-67. The positive cells were identified based on a threshold value. At least 500 cells were analyzed for each condition.

### 2.7. Kinase Inhibitors

The kinase inhibitors palbociclib, ribociclib, and abemaciclib used in this experimental setup are clinically applied in the treatment of estrogen receptor (ER)-positive, HER2-negative breast cancer [9,14,16]. These drugs are administered orally and are primarily metabolized hepatically via CYP3A4 [9,14]. The structural formulas of the kinase inhibitors used are shown in Figure 1A.

All three belong to the class of selective CDK4/6 inhibitors, as their mechanism of action is based on the inhibition of CDK 4 and 6, which have a lower side-effect profile than non-selective CDK inhibitors [1,9,14,16]. Upon binding to cyclin D, CDK4/6 forms a functional complex that phosphorylates the retinoblastoma protein (pRB), thereby inactivating it [9,14,16]. This inactivation leads to the release of the transcription factor E2F, which subsequently induces the expression of genes essential for cell cycle progression from G0-phase to G1-phase [9,14,16,17] (Figure 1B,C).

### 2.8. Statistics

Statistical analyses were performed using the one-tailed Mann–Whitney U test with a significance level of *p* < 0.05 (*n* = 3) for senescence, cell-cycle, and apoptosis/necrosis data. Correlations were evaluated by linear regression analysis, and the coefficient of determination (R^2^) was calculated for each fit. Immunostaining experiments were performed once (*n* = 1); therefore, no statistical analysis was applied.

The creation of graphs was performed using GraphPad Prism 9 (Graphpad Software Inc., Solana Beach, CA, USA). Images were analyzed using Biomas CountEasy3F (V 2.1). The schematic illustrations were created in BioRender, and the figures were compiled using Gimp (V 3.0.6).

## 3. Results

Our goal was to investigate the occurrence of senescence compared to cell death in eight breast cancer cell lines and two normal tissue cell lines when three different CDK4/6 inhibitors were combined with ionizing radiation.

### 3.1. Treatment with CDK Inhibitors Increases Cellular Senescence in MCF-7 and MDA-MB-231

**Figure 2:**
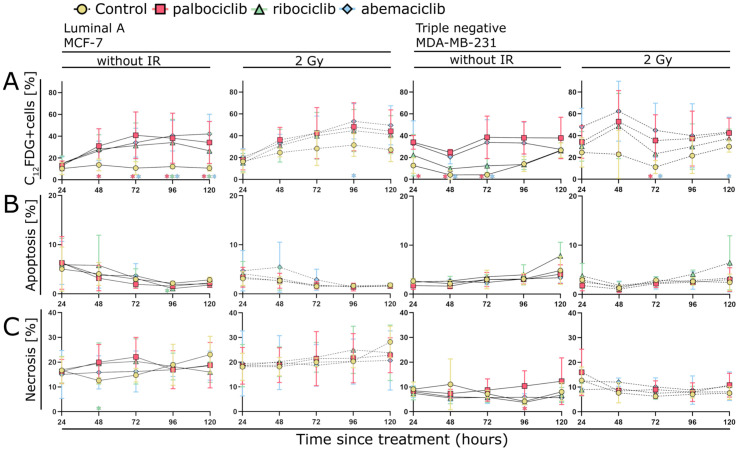
Senescence (C_12_FDG-positive cells) (**A**), apoptosis (Annexin V-positive, 7-AAD-negative) (**B**) and necrosis (Annexin V-positive, 7-AAD-positive) (**C**) of the MCF-7 and MDA-MB-231 cell lines were measured 24, 48, 72, 96 and 120 h after inhibitor addition. Cells were treated with 0.5 µM palbociclib, ribociclib, or abemaciclib with or without irradiation with 2 Gy 3 h afterwards. The non-drug-treated sample is represented by the yellow circle. Treatment with palbociclib is represented by the red square, treatment with ribociclib by the green triangle, and treatment with abemaciclib by the blue diamond. Solid lines indicate graphs without irradiation, whereas dashed lines indicate graphs with 2 Gy irradiation. Statistical significance was assessed using the one-tailed Mann–Whitney test with a significance level of *p* < 0.05. Each value represents the mean ± standard deviation (SD) (*n* = 3). Standard deviations are shown as color-coded error bars matching the color of each data point. The asterisks in the same color as the data point indicate its significance between the control and CDK inhibitor-treated samples (without IR) or between the irradiation monotherapy and the combined treatment (2 Gy).

Initially, we determined the change in senescence and cell death in MCF-7 and MDA-MB-231 cell lines over a period of 5 days (Figure 2). Treatment was carried out on day 1 using palbociclib, ribociclib, or abemaciclib with a target final dose of 0.5 µM, and partial irradiation with a single dose of 2 Gy. The samples were measured 24, 48, 72, 96, and 120 h after inhibitor addition. We identified senescence by staining with C_12_FDG and defined C_12_FDG-positive cells as senescent. Cell death was measured using Annexin V and 7-AAD staining, differentiated into apoptosis and necrosis, whereby we evaluated Annexin V-positive but 7-AAD-negative cells as apoptotic and Annexin V- and 7-AAD-positive cells as necrotic (Figure 2B,C). In summary, there was a distinct increase in the senescent population when treated with CDK inhibitors and/or irradiation in both MCF-7 and MDA-MB-231 cells (Figure 2A).

Treatment with CDK inhibitor alone showed a clear increase in senescence in the MCF-7 cell line over the entire measurement period of 5 days (Figure 2A). A statistically significant rise was observed for palbociclib after 48 h, for both palbociclib and abemaciclib after 72 h, and for palbociclib, ribociclib, and abemaciclib after 96 h and 120 h (Figure 2A). Irradiation alone also led to a noticeable increase in the senescent cell fraction, reaching a maximum around 48 h followed by only a slow further increase up to 96 h, with a significant effect detected for the combination of abemaciclib with 2 Gy at 96 h (Figure 2A). A slight additive tendency was observed with combined drug and radiation therapy, which was particularly evident after 96 and 120 h (Figure 2A). Senescence reached a plateau in the treated samples between 96 and 120 h, with a tendency to decline towards the last measurement point (Figure 2A). In contrast, the proportion of the senescent population in the control group remained consistent throughout the entire measurement period (Figure 2A).

In the MDA-MB-231 cell line, the senescent population increased clearly following treatment with CDK inhibitors and/or irradiation (Figure 2A). Initially, senescence decreased in all non-irradiated samples between 24 and 48 h, followed by a renewed increase (Figure 2A). Treatment with CDK inhibitors alone led to a distinct increase in senescence compared to the control group up to 72 h after treatment (Figure 2A). A statistically significant increase was observed for palbociclib at 24 h, and for both palbociclib and abemaciclib at 48 h and 72 h compared to the control group (Figure 2A).

After 96 and 120 h, the senescent population in the control also increased (Figure 2A). In addition, there was a visible difference between the inhibitors used: palbociclib induced a higher percentage of senescent cells over the entire measurement period, followed by abemaciclib and finally ribociclib. Palbociclib and abemaciclib reached a plateau after 72 h, but ribociclib showed an earlier plateau between 48 and 72 h, followed by a renewed increase after 120 h.

By contrast, the combination treatment resulted in a pronounced increase in the senescent population after 48 h, whereas the irradiated sample that was not treated with medication did not increase the proportion of senescent cells. After reaching a peak at 48 h with a senescence proportion of up to 60% with abemaciclib and 2 Gy, there was a marked decline at 72 h, followed by a slight upward trend until the end of the measurement period. A statistically significant difference between irradiation alone and the irradiated samples treated with CDK inhibitors was observed for palbociclib and abemaciclib at 72 h, and for abemaciclib at 120 h (Figure 2A).

In combination therapy, abemaciclib produced more senescence on average than palbociclib. Ribociclib achieved similarly high proportions of senescent cells as palbociclib in combination with irradiation.

### 3.2. CDK Inhibitors Show Little Effect on Cell Death in MCF-7 and MDA-MB-231

The values for apoptosis and necrosis remained constant in both cell lines throughout the entire measurement period (Figure 2B,C). MCF-7 cells exhibited more necrosis than apoptosis. The proportion of apoptotic cells decreased over the measurement period, whereas the proportion of necrotic cells slightly increased (Figure 2B,C). There was no clear effect of the treated samples compared to the control (Figure 2B,C). A higher proportion of necrotic than apoptotic cells were present in the MDA-MB-231 cell line, though this difference was smaller than that observed in the MCF-7 cell line (Figure 2B,C). Here, too, the apoptosis and necrosis values remained relatively constant over the measurement period, and again there were no deviations in the treated samples compared to the control (Figure 2B,C).

### 3.3. Effect of CDK Inhibition on Senescence in All Cell Lines 72 H After Inhibitor Addition

**Figure 3:**
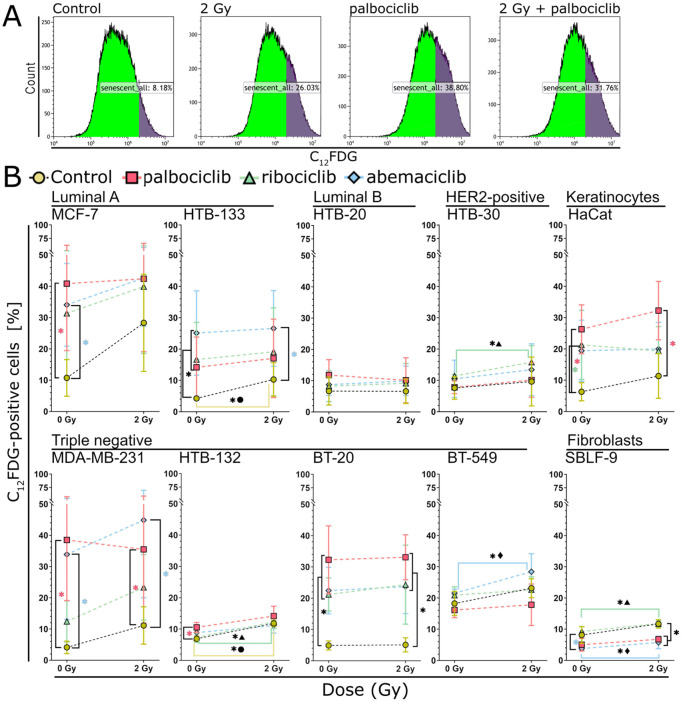
Occurrence of senescence in eight breast cancer cell lines and two healthy cell lines 72 h after treatment with 0.5 µM palbociclib, ribociclib, or abemaciclib with or without an additional irradiation with 2 Gy 3 h afterwards. (**A**) Example of the gating strategy for determining senescence using Kaluza Analysis Software based on MCF-7 cell line. The dark area indicates the region containing primarily senescent cells. (**B**) The percentage of cells undergoing senescence induction in all ten cell lines examined 72 h after inhibitor addition. We defined C_12_FDG-positive cells as senescent. The non-drug-treated sample is represented by the yellow circle. Treatment with palbociclib is represented by the red square, treatment with ribociclib by the green triangle, and treatment with abemaciclib by the blue diamond. The scale of the graphs was chosen to be uniform for better comparison. Significance was determined using a one-tailed Mann–Whitney U test with *p* < 0.05. Each value represents the mean ± standard deviation (SD) (*n* = 3). Standard deviations are shown as color-coded error bars matching the color of each data point. Vertical brackets show significance within irradiation groups: control vs. treatment with CDK inhibitor at 0 Gy; irradiation monotherapy vs. combined treatment at 2 Gy. Asterisks are color-matched to treatments (red: palbociclib; green: ribociclib; blue: abemaciclib; black: more than one of the conditions listed above). Horizontal brackets indicate significance between irradiation groups (0 Gy vs. 2 Gy), color-coded as above (red: palbociclib; green: ribociclib; blue: abemaciclib). Symbols accompany asterisks for clarity (circle: non-drug-treated samples; triangle: ribociclib; diamond: abemaciclib).

After determining a useful timepoint for the measurement of senescence, we performed the experiment described previously on six additional breast cancer cell lines and two healthy cell lines. The gating strategy to define C_12_FDG-positive cells (Kaluza Analysis Software) was established individually for each cell line based on the controls and is shown for the breast cancer cell line MCF-7 as an example (Figure 3A).

There were marked differences in the induction of senescence by ionizing radiation and the CDK inhibitors (Figure 3B). Irradiation had no effect on BT-20 and HTB-20 cells, but increased senescence in all other cell lines, reaching a peak in MCF-7 cells, where senescence increased by 15% due to irradiation alone. Similarly, the three CDK inhibitors had little to no effect when administered alone on HTB-20, HTB-30, HTB-132, BT-549, and SBLF-9. In the remaining cell lines MCF-7, HTB-133, HaCaT, MDA-MB-231, and BT-20, however, the inhibitors substantially induced senescence compared to control, reaching 40% in MCF-7 cells when treated with palbociclib. This increase in senescence was significant in the MCF-7 and MDA-MB-231 cell lines between the control group and the samples treated with palbociclib and abemaciclib. In the HBT-133 and BT-20 cell lines, the increase in senescence between the control group and all inhibitor-treated samples was also significant. In the HaCaT cell line, a significant increase in senescence was observed between the control group and the samples treated with palbociclib and ribociclib. Combining the inhibitors with irradiation led to a slightly additive effect in some cell lines, such as in MCF-7 and MDA-MB-231, for example, when treated with ribociclib and abemaciclib; however, these changes were not statistically significant. Significant changes could be observed in HTB-30, HTB-132 and SBLF-9 for ribociclib as well as in BT-549 and SBLF-9 for abemaciclib. The three CDK inhibitors exhibited varying degrees of senescence depending on the cell line, and it was not possible to identify the most effective inhibitor.

### 3.4. Comparison Between p21 and C_12_FDG Staining: Most Cell Lines Show Similar Trends

**Figure 4:**
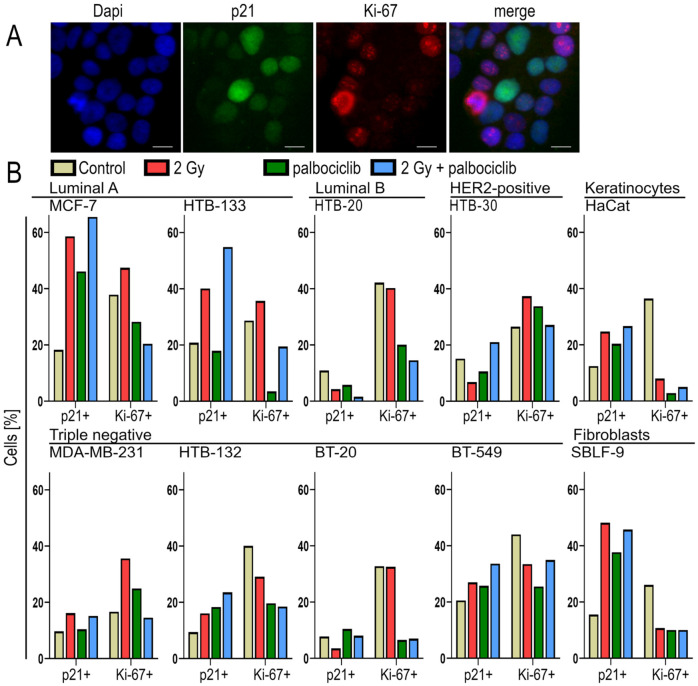
Determination of senescence by immunostaining of p21. All ten cell lines were treated with 0.5 µM of the CDK inhibitor palbociclib and 2 Gy ionizing radiation 3 h afterwards. Cells were stained with anti-p21 as a senescence marker and anti-Ki-67 as a proliferation marker. (**A**) Representative microscopic images after immunostaining, using the HTB-132 cell line as an example. DAPI (blue), p21 (green), and Ki-67 (red) are shown separately and then superimposed at the end. Scale bar represents 10 µm. (**B**) Proportion of p21-positive senescent cells and proportion of Ki-67-positive proliferating cells after irradiation, treatment with palbociclib and the combination. The untreated sample is represented by the yellow bar, while the 2 Gy irradiation is represented by the red bar. The green bars represent treatment with palbociclib, and the blue bars represent combined treatment. *n* = 1.

We performed a further determination of senescence with immunostaining of p21 using the same experimental procedure, again measuring 72 h after inhibitor addition on all 10 cell lines. The primary goal of this experiment was to provide a visual representation and to assess the overall trend rather than to perform a quantitative analysis; consequently, the experiments were performed only once. Palbociclib was randomly selected as the representative CDK inhibitor. The examination was performed on all ten cell lines, but only once in each case. The measured values are therefore considered less valid than the results of flow cytometry and are therefore only evaluated in terms of their tendency. Representative microscopic images are shown as an example using cell line HTB-132 in Figure 4A. The graphs in Figure 4B were created by quantifying the radiance of the fluorescence signal of the nuclei.

Similar trends were evident in the various test conditions when measuring senescence using both methods, particularly in the HTB-132, BT-549, HTB-20 and HTB-30 cell lines, and in the HaCaT keratinocytes (Figure 4B). These cell lines mostly presented a similar level of the senescent population in both measurement methods. The MCF-7 and HaCaT cell lines showed comparable trends using both methods, but the senescent population was larger when measured using p21. The same was true of the SBLF-9 fibroblasts. By contrast, significantly smaller senescent populations were observed in the MDA-MB-231, HTB-133 and BT-20 cell lines when measured using p21 compared to C_12_FDG. 

### 3.5. Palbociclib Effectively Lowers Proliferation Index

Measurement of the proliferation index using Ki-67 revealed a decreasing trend in both healthy cell lines regardless of treatment; the use of palbociclib or irradiation consistently resulted in a substantial reduction (Figure 4B). Measurement of the proliferation index using Ki-67 revealed that, in the healthy cell lines (fibroblasts SBLF-9 and keratinocytes HaCaT), there was a consistent reduction with all treatments. This trend was also observed in breast cancer cell lines, albeit to a lesser extent in HTB-132 and BT-549. In most malignant cell lines, a slight increase (MCF-7, HTB-133, HTB-30 and MDA-MB-231) or stabilization (HTB-20 and BT-20) was observed following irradiation at 2 Gy. Only when the CDK inhibitor palbociclib was added was a reduction in Ki-67 expression observed.

### 3.6. Necrosis as the Main Pathway of Cell Death in All Cell Lines Examined

**Figure 5:**
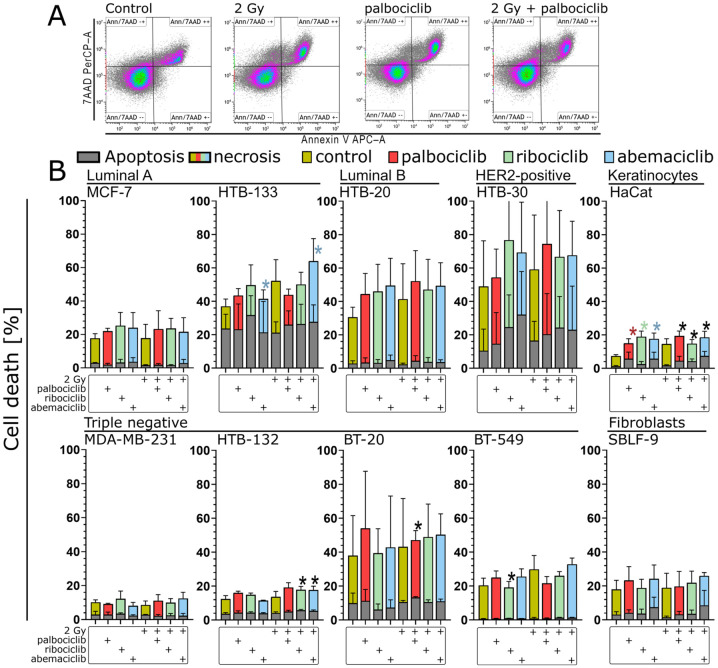
Overview of cell death, divided into apoptosis and necrosis, in all ten cell lines examined. (**A**) Gating strategy using Kaluza Analysis Software based on the MCF-7 cell line as an example. Color intensity indicates event density. Here, too, gating was performed individually for each cell line based on the controls. (**B**) Cell death in all cell lines. Treatment matrix showing the presence (+) of 2 Gy irradiation, palbociclib, ribociclib, and abemaciclib for each bar. Apoptosis is always shown in gray, while necrosis was additionally color-coded for each test condition (yellow: non-drug-treated sample; red: palbociclib; green: ribociclib; blue: abemaciclib). The scale of the graphs was chosen uniformly here for better comparability. We defined the apoptotic population as Annexin V-positive but 7-AAD-negative, and the necrotic population as Annexin V- and 7-AAD-positive. Statistical analysis was performed using a one-tailed Mann–Whitney U test with a significance level of *p* < 0.05. Each value represents the mean ± standard deviation (SD) (*n* = 3). For necrosis, color-coded asterisks above bars indicate statistical significance between control and CDK inhibitor-treated samples, and between irradiation monotherapy and combined treatments (red: palbociclib; green: ribociclib; blue: abemaciclib). Black asterisks above bars indicate significance for the same comparisons in apoptosis.

In addition to senescence, cell death, such as apoptosis and necrosis, also leads to permanent cell inactivation. Necrosis and apoptosis were measured by flow cytometry using Annexin V and 7-AAD staining. Apoptotic cells were defined as Annexin V-positive and 7-AAD negative, whereas necrotic cells were defined as Annexin V- and 7-AAD positive (Figure 5A). In most cell lines, the proportion of apoptotic cells was either negligible (BT-549) or only slightly increased, regardless of treatment (MDA-MB-231, HTB-132 and MCF-7) (Figure 5B). In some cell lines, however, there was a slight increase compared to the control. In particular, the cell lines HTB-30, BT-20 and HTB-133 showed a higher background of apoptotic cells, in BT-20 and HTB-133, independent of treatment. Only in the HTB-30 cell line was there an increase in apoptosis due to the CDK inhibitors, though there was no additive effect when they were combined.

The proportion of necrosis was greater than that of apoptosis in all cell lines and for each test condition (Figure 5B). Irradiation alone had no effect on total cell death in the MDA-MB-231, HTB-132 and SBLF-9 cell lines, and treatment with CDK inhibitors also had no additional effect in these cell lines. In the MCF-7 cell line, irradiation did not trigger additional cell death, but the CDK inhibitors did. In the remaining cell lines, irradiation and the CDK inhibitors both triggered a slight increase in cell death, mainly through an increase in necrosis. The combination had no additive effect in any of the cell lines (Figure 5B).

### 3.7. Changes in G2 Arrest Under CDK4/6 Inhibition and Radiation

**Figure 6:**
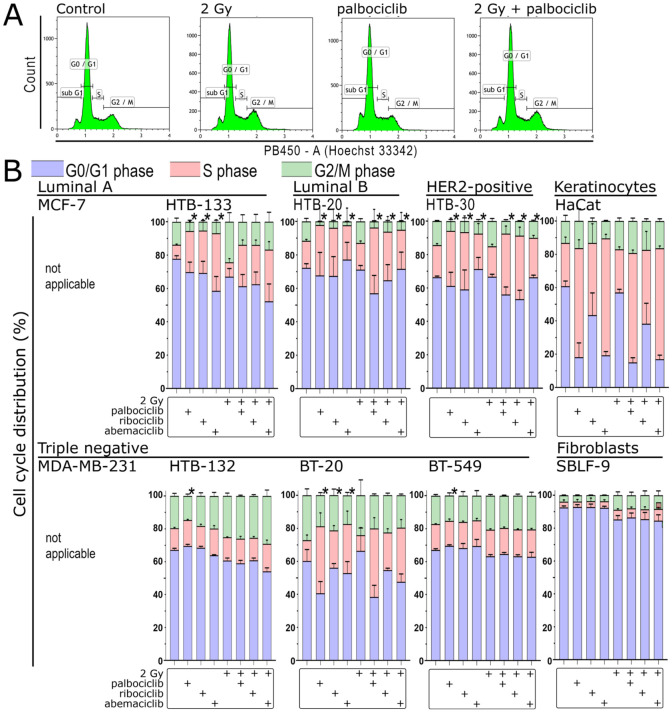
Cell cycle distribution in eight cell lines after irradiation, treatment with the CDK inhibitors palbociclib, ribociclib and abemaciclib, and combination with ionizing radiation. (**A**) Cell cycle gating strategy using Kaluza Analysis Software, based on the breast cancer cell line HTB-132 as an example. (**B**) Individual cell cycle phases for eight cell lines. The MCF-7 and MDA-MB-231 cell lines are excluded due to multidrug resistance, which prevents live staining with Hoechst 33342 and subsequent cell cycle analysis. Treatment matrix showing the presence (+) of 2 Gy irradiation, palbociclib, ribociclib, and abemaciclib for each bar. Statistical analysis was performed using a one-tailed Mann–Whitney U test with a significance level of *p* < 0.05. Each value represents the mean ± standard deviation (SD) (*n* = 3). Statistically significant differences in the proportion of G2 arrest between the control and CDK inhibitor treatment, and between irradiation monotherapy and combined CDK inhibitor and irradiation, are shown by black asterisks above each bar.

Since G2 arrest is the most sensitive phase of the cell cycle to irradiation in the fractionated treatment of cells, we next examined the cell cycle distribution after our treatment protocol. Cell-cycle dependency was measured using Hoechst 33342 staining and cytometry (Figure 6A). Irradiation alone did not affect G2 arrest in the HTB-20, HTB-30, BT-20, BT-549 and HaCaT cell lines, but it did affect it in the HTB-133 cell line by increasing the proportion of cells in G2/M (Figure 6B). In HTB-20, HTB-30, BT-20 and HTB-133 cell lines, G2 arrest decreased with both single and combination treatments. In BT-549 and HaCaT, the proportion of G2 arrest remained constant with both inhibitors and with the combination. In HTB-132 and SBLF-9, the combination produced a similar increase to that seen with irradiation alone.

### 3.8. A Comparison of the Occurrence of Senescence and Cell Death Following Various Treatments in Ten Cell Lines

**Figure 7:**
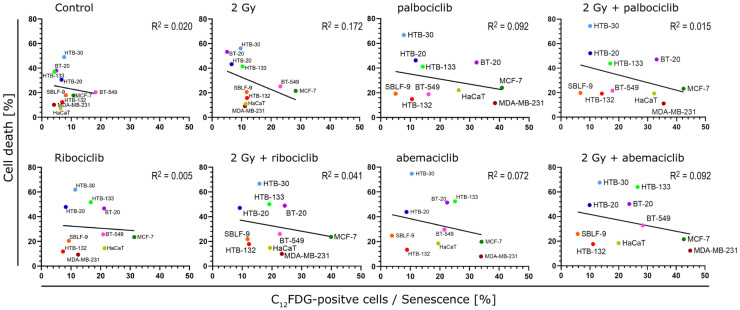
Correlation between senescence on the abscissa and cell death on the ordinate for each cell line under the different conditions. Each graph represents an experimental setting, with the ten cell lines color-coded and labelled accordingly. Data were analyzed using linear regression with the coefficient of determination (R^2^) reported for each fit.

Examining the proportion of cell lines that undergo senescence or cell death throughout the plots reveals that HTB-132, HTB-30, HTB-20 and SBLF-9 deviate minimally, indicating a slight response to the administered treatment (Figure 7). Cell death clearly dominates in the HTB-30 and HTB-20 cell lines, with only a moderate proportion of senescent populations; however, the treatment has little overall effect on cell death. By contrast, HTB-132 and SBLF-9 remain stable and demonstrate minimal cell death and senescence in all conditions. The MDA-MB-231, MCF-7, BT-549 and HaCaT cell lines had a clear tendency to form senescence and exhibited little cell death. BT-20 and MDA-MB-231 cell lines clearly demonstrate the effect of CDK inhibitors on senescence; both cell lines strongly promote the formation of senescent populations as soon as treatment with a CDK inhibitor is administered. In contrast, irradiation alone had no effect on the senescent population.

**Figure 8:**
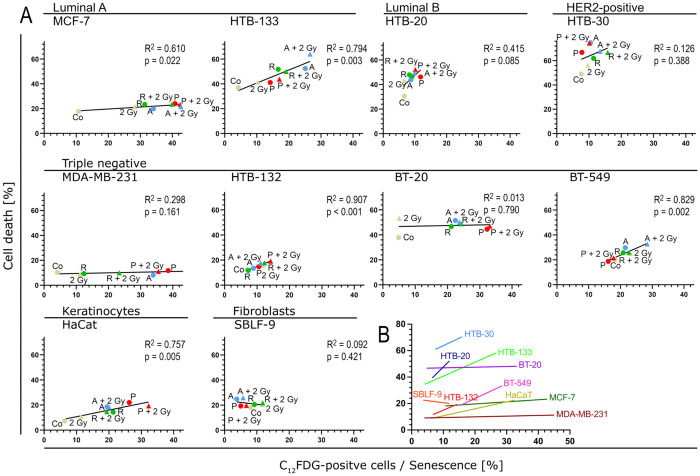
Relationship between senescence and cell death. (**A**) Correlation between senescence on the abscissa and cell death on the ordinate is shown for each cell line individually. Experimental conditions are labeled and color-coded consistently with previous figures (non-drug-treated samples: yellow; palbociclib: red; ribociclib: green; abemaciclib: blue). In addition, different symbols mark the irradiated (triangle) and non-irradiated (circle) samples. (**B**) Overlay of the regression lines of all graphs shown in A. Co = control, 2 Gy = dose of 2 Gy ionizing radiation, P = palbociclib, R = ribociclib, A = abemaciclib. Data were analyzed using linear regression. The coefficient of determination (R^2^) is reported for each fit, and the *p*-value indicates whether the slope is significantly different from zero.

In most cell lines, we observed a marked increase in the senescent population with a simultaneous escalation of the therapies used, whereas an increase in cell death was mostly only minor (Figure 8A). The BT-20, MDA-MB-231, and MCF-7 cell lines responded to therapy almost exclusively by forming senescence, with cell death being nearly negligible in this case. The cell lines BT-549, HTB-133, HTB-30, and HaCaT also showed a stronger tendency to form senescence than to present cell death. The cell line HTB-20 expressed the strongest tendency to form cell death, but even here an increase in the senescent population can be observed. 

When all regression lines are overlaid, it becomes clear that the changes in cell death and senescence vary greatly in their extent (Figure 8B). The cell lines HTB-30, HTB-20, SBLF-9, and HTB-132 show a lower response to therapy compared to the cell lines MDA-MB-231, MCF-7, BT-20, BT-549, and HaCaT. In summary, it can be added that no cell line showed a comparable strong tendency to form cell death as to form senescence. Furthermore, no CDK inhibitor can be identified here that clearly outperforms the others in terms of efficacy.

## 4. Discussion

In the cell lines examined, there were very large interindividual differences between the cell lines in terms of the occurrence of cell death and senescence. In the eight breast cancer cell lines, senescence was mostly induced after treatment with CDK inhibitors, whereas cell death responded only slightly to the treatment in most cell lines. Combined treatment consisting of CDK inhibition and irradiation induced a slightly additive effect in the time window examined in some cell lines, but no additive or superadditive effect. 

In the MCF-7 and MDA-MB-231 cell lines, senescence induced by CDK inhibition was most pronounced, whereas apoptosis and necrosis values fluctuated less strongly across the different experimental conditions, suggesting that cell inactivation in these cell lines is primarily due to senescence rather than cell death. In contrast, the HTB-132 and SBLF-9 cell lines showed little senescence formation and little cell death across all experimental conditions. Necrosis was the main form of cell death in all cell lines examined. Only a few cell lines (HTB-30, BT-20, and HTB-133) showed pronounced apoptotic populations, but only the HTB-30 cell line showed an increase after treatment with CDK inhibition; nevertheless, necrosis was the main form of cell death in this case as well.

Various preclinical studies have already described the tendency for senescence to develop in many breast cancer cell lines following CDK4/6 inhibition [18,19,20,21,22,23]. In other studies, the breast cancer cell line BT-546 also showed no or only reduced senescence formation after CDK inhibition, which was interpreted as resistance [22].

Cell death, especially apoptosis, did not appear to be the main mechanism of action following CDK inhibition in breast cancer cells, as we have previously described [24] and as has also been found in other studies [25]. In some cases, resistance to the development of apoptosis in senescent tumor cells has even been described [22,26,27], but targeted induction of apoptosis can be used as an anti-tumor strategy [28,29]. For example, apoptosis in MDA-MB-231 was achieved by a combination treatment of abemaciclib and ABT-263 [19].

Our data did not show a clear additive effect after irradiation and CDK inhibition. However, a radiosensitive effect after combined treatment with CDK inhibition and irradiation is described in the literature for various cell lines [30,31]. Leading examples here are cell lines from brain tumors (glioblastoma, medulloblastoma), lung cancer (small cell and non-small cell carcinomas), colorectal cancer, and head and neck squamous cell carcinomas [30,31]. However, this effect has also been described in isolated cases for breast cancer cell lines [32]. An increase in the additive effect appears to be possible in some cases by changing the order of the treatment sequence, for example, if radiation is administered before CDK inhibition [33].

From a clinical perspective, an additive effect of both treatments can lead to an increase in side effects, but current studies indicate that this is not generally to be expected [3]. Even though there are individual case reports of severe side effects in the skin tissue or gastrointestinal tract [34], most and larger studies suggest manageable side effects [3].

The determination of senescence using C_12_FDG and p21 as senescence markers showed similar trends in both methods within the individual cell lines, with the change in senescence in response to the various treatment options being particularly similar. However, the extent of senescence formation differed, with some cell lines showing more senescence in the p21 measurement (MCF-7, HaCaT), but others showing smaller senescent populations according to this measurement method than in the C_12_FDG measurement (MDA-MB-231, HTB-133, BT-20). But these are two different markers for senescence and therefore represent different aspects of senescence. P21 is considered a marker of the p53-dependent senescence program and induces a stable and permanent cell cycle arrest, whereas C_12_FDG is a substrate for acidic β-galactosidase, which is directly related to senescence and is therefore considered a very reliable marker for senescence [35,36,37].

As a representative of CDK inhibitors, palbociclib showed a clear reduction in the proliferation index in all cell lines, whereas irradiation alone led to an increase in the proliferation index in most breast cancer cell lines. The situation was different in healthy cell lines SBLF-9 and HaCaT, where a marked reduction was always observed regardless of the therapy administered. These also responded to treatment with only minimal cell death, and there was also little evidence of senescence, particularly in the fibroblasts. There were hardly any additive effects with combined treatment, not even in the cell cycle analysis. The examination of the proliferation marker confirmed this. Although all treatments led to a reduction in marker expression by at least half, further escalation of therapy showed no additive effect. This suggests that there is a therapeutic window for effective treatment of tumor cells without increasing damage to healthy tissue. Radio-adaptive response has been described several times in the literature. In this case, low-dose radiation leads to a change in gene expression, so that subsequent therapies are less damaging to cells [7,38].

The groupings that the breast cancer cell lines showed under the various experimental conditions varied from the subtypes listed in Table 1. The classification within these subtypes (luminal A, luminal B, HER2-positive, triple-negative) therefore did not allow any predictive statements to be made about the effect of the various experimental conditions on senescence, cell death, or the cell cycle. Given that breast cancer is highly heterogeneous and that classification can vary greatly depending on the literature, it is not surprising that the classification most commonly used clinically cannot be reproduced at the cellular level, but that functional characteristics such as senescence, cell death, and cell cycle behavior can vary considerably within a subtype [7]. 

Premature senescence occurs in both tumor tissue and normal tissue and must therefore be considered separately for each [39,40]. In tumors, therapy-induced senescence of tumor cells will lead to increased efficacy [21,41]. But it also means that an increasing number of senescent tumor cells remain, posing a risk of late recurrence [21]. For these cells, the idea has emerged that senescent cells represent a specific target for drugs known as “senolytics,” which are designed to eliminate them in a targeted manner [21]. The main advantage of senescence in normal tissue is that there is no cell loss in the tissue; instead, cells that are no longer capable of division remain present, thereby preventing the damage caused by cell loss, as occurs in necrotic cell death, resulting in the formation of necrotic areas [42,43]. However, because increased senescence is induced in tissue, it is assumed that senescence induces a pro-inflammatory environment, the SASP [5,6,16,44,45,46]. In this context, a stronger SASP environment is expected for chemotherapy and radiation therapy [47], whereas for CDK4/6 inhibitors a weaker SASP environment is assumed, though with increased expression of immunosuppressive ligands [48]. This in turn carries the problem that the inflammation increases the risk of tumor recurrence or secondary carcinomas as well as the risk of fibrosis [16,45,46,49].

## 5. Conclusions

Treatment with the CDK inhibitors palbociclib, ribociclib and abemaciclib did induce senescence in many breast cancer cell lines, particularly evident in MCF-7 and HTB-133, which are ER- and PR-positive and HER2-negative and thus represent the subtype currently targeted by CDK4/6 inhibitors in clinical practice, as well as in the triple-negative cell lines MDA-MB-231 and BT-20, which are not routinely treated with CDK4/6 inhibitors, but still showed a marked increase in senescent cell populations [1,2,7,8]. Cell death, on the other hand, was not a main cell fate after CDK inhibition. Combination therapy with irradiation showed at most a minor additive effect on senescence and cell death. Therefore, senescent cells could occur more frequently in tumor therapy, thereby increasing the risk of senescence-associated complications. These include increased inflammation and fibrosis. Furthermore, no CDK inhibitor can be identified that clearly outperforms the others in terms of efficacy.

In future studies, the next step could involve investigating additional HR-positive and HER2-negative breast cancer cell lines—that is, the molecular subtype for which current clinical guidelines recommend CDK inhibition. Furthermore, the underlying mechanisms that lead to senescence induction in the various subtypes could be examined in greater detail. For example, by identifying molecular markers that may also allow for predictions about different cell fates. Studies that examine the effects of senolytic drugs within a similar research framework—drugs that could potentially reduce side effects—also represent a promising approach. Similarly, research that examines the timing of CDK inhibition and radiotherapy in greater detail appears promising and may be of direct clinical relevance.

## Figures and Tables

**Table 1:** The table provides an overview of the cell lines used, alternative names, and molecular cell status, adapted from [7,8]. In addition, the histological classification into invasive ductal carcinoma (IDC) and adenocarcinoma (AC) is indicated [7,8].

Cell Lines	ER	PR	HER2	Tumor Subtype
**Luminal A**				
MCF-7 (HTB-22)	+	+	−	IDC
HTB-133 (T-47D)	+	+	−	IDC
**Luminal B**				
HTB-20 (BT-474)	+	+	+	IDC
**HER2-positive**				
HTB-30 (SK-BR-3)	−	−	+	AC
**Triple-Negative**				
MDA-MB-231 (HTB-26)	−	−	−	AC
HTB-132 (MDA-MB-468)	−	−	−	AC
BT-20 (HTB-19)	−	−	−	IDC
BT-549 (HTB-122)	−	−	−	IDC

**Table 2:** Serum levels in patients following administration of palbociclib, ribociclib, and abemaciclib. Where available, steady-state mean serum concentrations were reported; otherwise, the minimum and maximum concentrations observed were reported.

Drug	Dosage	Plasma Concentration in µM
Palbociclib [11]	125 mg daily for 3 weeks	Mean: 0.415
Ribociclib [12]	900 mg daily for 5 days	Mean: 1.496 ± 0.674
Abemaciclib [13]	200 mg twice daily	min: 0.389, max: 0.588

**Table 3:** Detailed overview of the individual experimental days.

Time	Action	Time After Treatment
**Day 0**	Cell seeding	
**Day 1**	Treatment with inhibitor and/or irradiation	
**Day 2**	Flow cytometry analysis: MCF-7 and MDA-MB-231	24
**Day 3**	Flow cytometry analysis: MCF-7 and MDA-MB-231	48
**Day 4**	Flow cytometry analysis: All cell lines	72
**Day 5**	Flow cytometry analysis: MCF-7 and MDA-MB-231	96
**Day 6**	Flow cytometry analysis: MCF-7 and MDA-MB-231	120

## Data Availability

The data presented in this study are available upon request from the corresponding author.

## Supplementary Materials

The following supporting information can be downloaded at: https://www.mdpi.com/article/10.3390/cells15080734/s1. Figure S1: Gating strategy for C_12_FDG, apoptosis/necrosis, and cell cycle distribution. Abemaciclib-treated BT-549 and HTB-132 are shown representatively. Initially, cell debris and doublets were excluded. Afterwards, thresholds for C_12_FDG, Annexin V, and 7-AAD were defined, and the cell cycle phases were distinguished according to their DNA content and corresponding Hoechst 33342 signal.

## Abbreviations

The following abbreviations are used in this manuscript:

AC	Adenocarcinoma
BAF	Bafilomycin A 1
CDK	Cyclin-dependent kinase
DMEM	Dulbecco’s Modified Eagle’s Medium
DMSO	Dimethyl sulfoxide
DPBS	Dulbecco’s Phosphate-Buffered Saline
ER	Estrogen receptor status
FBS	Fetal Bovine Serum
HER2	Human epithelial receptor 2
HR	Hormone receptor status
PR	Progesterone receptor status
IDC	Invasive ductal carcinoma
NEA	Non-essential amino acids
OIS	Oncogene-induced senescence
pRB	Retinoblastoma protein
SA-β-GAL	Senescent-associated β-galactosidase
SASP	Senescence-associated secretory phenotype
TBS	Tris-buffered saline
