# Supplementary material for: Combined CDK4/6 Inhibition and Radiation: Effects on Cellular Senescence, Cell Cycle Regulation, and Cell Death in Mammary Carcinoma Cells
Source: Cells. 2026 Apr 21;15(8):734. doi: 10.3390/cells15080734 (PMC13114986; doi:10.3390/cells15080734)
Supplement: Supplementary file 1 [file cells-15-00734-s001.zip › cells-4226362-supplementary.pdf]

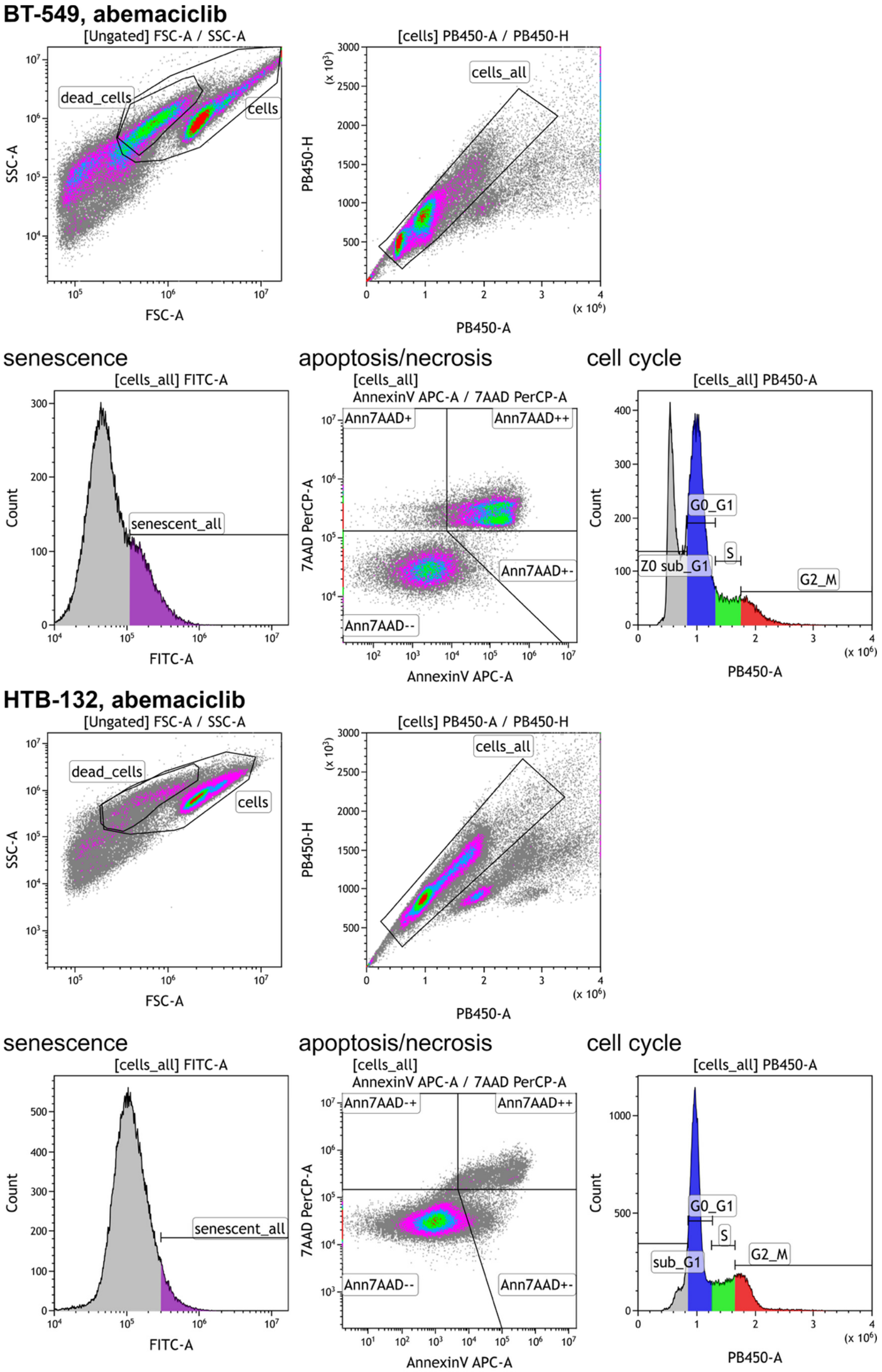

Figure S1: Gating strategy for $C_{12}FDG$, apoptosis/necrosis, and cell cycle distribution. Abemaciclib-treated BT-549 and HTB-132 are shown representatively. Initially, cell debris and doublets were excluded. Afterwards, thresholds for $C_{12}FDG$, Annexin V, and 7-AAD were defined, and the cell cycle phases were distinguished according to their DNA content and corresponding Hoechst 33342 signal.
